# Comparison of Safety and Insurance Payments for Minor Hand Procedures Across Operative Settings

**DOI:** 10.1001/jamanetworkopen.2020.15951

**Published:** 2020-10-13

**Authors:** Jessica I. Billig, Jacob S. Nasser, Jung-Sheng Chen, Yu-Ting Lu, Kevin C. Chung, Chang-Fu Kuo, Erika D. Sears

**Affiliations:** 1 Veterans Affairs (VA)/National Clinician Scholars Program, VA Center for Clinical Management Research, VA Ann Arbor Healthcare System, Ann Arbor, Michigan; 2 Section of Plastic Surgery, Michigan Medicine, Ann Arbor; 3 Medical student, George Washington School of Medicine, Washington, DC; 4 Center for Artificial Intelligence in Medicine, Division of Rheumatology, Allergy and Immunology, Chang Gung Memorial Hospital, Taoyuan, Taiwan; 5 Department of Rheumatology, Allergy and Immunology, Chang Gung Memorial Hospital, Taoyuan, Taiwan; 6 Department of Rheumatology, Orthopaedics, and Dermatology, School of Medicine, University of Nottingham, Nottingham, United Kingdom; 7 VA Center for Clinical Management Research, VA Ann Arbor Healthcare System, Ann Arbor, Michigan

## Abstract

**Importance:**

Surgical procedures can be performed in different settings, but the association between the operative setting and patient safety and cost to the patient and payer is unknown.

**Objective:**

To examine differences in complications, total payments, and out-of-pocket (OOP) spending for minor hand surgical procedures performed in office, ambulatory surgery center (ASC), and hospital outpatient department (HOPD) operative settings.

**Design, Setting, and Participants:**

A retrospective, population-based cohort study was conducted using deidentified claims data from private employer-sponsored health insurance from January 1, 2009, to December 31, 2017. Patients aged 18 years or older undergoing carpal tunnel release, trigger finger release, excision of wrist ganglion, and excision of small hand masses (N = 468 365) were included.

**Exposures:**

Operative setting, defined as procedures performed in the clinic setting, ASC, and HOPD.

**Main Outcomes and Measures:**

Complications during the 90-day postoperative period, total payments (total facility and payer reimbursement), and OOP spending.

**Results:**

Of the 468 365 patients, 296 378 women (63.3%) and 171 987 men (36.7%) underwent minor hand surgical procedures from 2009 to 2017, with 284 889 procedures (60.8%) performed in HOPDs, 158 659 procedures (33.9%) performed in ASCs, and 24 817 procedures (5.3%) performed in the office setting. Ninety-day complications occurred in 3.4% of procedures performed in HOPDs, 3.3% in ASCs, and 2.9% in office settings (*P* < .001). After controlling for patient characteristics, procedures performed outside of the office had higher odds of complications (HOPDs: odds ratio [OR], 1.32; 95% CI, 1.22-1.43; ASCs: OR, 1.24; 95% CI, 1.14-1.34). Compared with the office setting, procedures performed in HOPDs incurred an extra $1216 in total payments (95% CI, $1184-$1248) and $115 in OOP expenses (95% CI, $109-$121). Procedures performed in ASCs cost an additional $709 (95% CI, $676-$741) and $140 in OOP expenses (95% CI, $134-$146). Transitioning ASC and HOPD procedures to the office setting could have saved an estimated $6 million annually in OOP expenses during the study period.

**Conclusions and Relevance:**

The findings of this study suggest that minor hand surgery performed in the office setting is safe and less costly compared with ambulatory and hospital-based operations. Shifting minor surgical procedures to the office setting may lead to substantial cost savings for payers and patients without compromising care quality.

## Key Points

**Question:**

What are the complications, total payments, and out-of-pocket expenses associated with minor hand surgical procedures performed in different operative settings?

**Findings:**

In this population-based cohort study of 468 365 minor hand surgical procedures, complications occurred in 3.4% of procedures performed in hospital outpatient departments, 3.3% in ambulatory surgery centers, and 2.9% in office settings. However, ambulatory and hospital-based procedures were significantly more expensive for payers and patients.

**Meaning:**

The findings of this study suggest that transitioning minor hand procedures to the office may reduce spending for payers and patients without compromising postoperative outcomes.

## Teaser

This cohort study examines the differences in cost among hospital outpatient departments, ambulatory surgery centers, and office settings for minor hand surgical procedures.

## Introduction

Advances in surgical technique, anesthesia, and medicine have permitted surgeons to perform procedures in different operative settings. Procedures once reserved solely for the hospital-based operating room are now being performed in ambulatory surgery centers (ASCs) and office settings. For minor surgical procedures, office-based surgery may be one avenue for cost savings. Specifically, in hand surgery, there has been a growing interest in performing hand procedures using the wide awake local anesthesia no tourniquet technique in the office. Retrospective single-institution studies have suggested that office-based procedures can lead to considerable cost savings.^1,2^ However, from a population-level standpoint, little is known regarding national spending and out-of-pocket (OOP) expenses for minor hand surgical procedures performed in different operative settings.

A major concern regarding transferring procedures from the operating room to the office is the potential risk of greater complications and poor outcomes. Several studies have assessed the perioperative and postoperative complications of transitioning inpatient procedures to ASCs with procedure-specific recommendations.^3,4,5^ However, there is a lack of data comparing outcomes and complication rates of procedures performed in hospital-, ambulatory-, and office-based operative settings. As health care expenditures continue to increase, with surgery accounting for approximately one-third of all health care spending,^6^ there is a need to identify strategies to decrease expenditures without compromising care quality.

Given the efforts to improve value in health care,^7^ further investigation is warranted to understand the association between operative setting and patient outcomes and spending for minor procedures. Therefore, the purpose of this study was to perform a population-based analysis of complication rates of minor hand procedures performed in different operative settings. In addition, we sought to investigate differences in total cost and OOP spending across different operative settings. Our goal was to report findings that may help inform policy with regard to high-quality and high-value surgical care.

## Methods

### Data Source and Cohort Selection

We performed a retrospective cohort study using data from the IBM MarketScan Research databases between 2009 and 2017. These databases contain information from more than 240 million patients enrolled in employer-sponsored health insurance from more than 350 payers nationwide and include longitudinal health care encounters, patient-level costs, and pharmaceutical data.^8^ The IBM MarketScan databases used in this analysis comprise the Commercial Claims and Encounters Database. This data source includes traditional employer-sponsored health insurance and the Medicare Supplemental and Coordination of Benefits Database, which contains Medicare supplemental insurance paid by employers. To obtain this cohort, we selected patients aged 18 years or older undergoing open carpal tunnel release, trigger finger release, excision of wrist ganglion cysts, or excision of hand masses using *Current Procedural Terminology* codes (eTable 1 in the Supplement). The procedures were chosen based on their low complexity and potential to be performed under local anesthesia in any operative setting (eg, hospital outpatient department [HOPD], ASC, and the office). Endoscopic carpal tunnel release was not included because it is not typically performed in all 3 operative settings under local anesthesia. We ensured specificity of the procedures by also associating each procedure with relevant *International Classification of Disease, Ninth, Clinical Modification* (*ICD-9*) or *International Statistical Classification of Diseases and Related Health Problems, Tenth Revision* (*ICD-10*) diagnosis codes (eTable 1 in the Supplement). All patients included in the cohort had continuous enrollment in the database 6 months before the surgery and 3 months after the surgery to permit observation of comorbidities and postoperative complications. We excluded patients who had procedures performed during an inpatient hospital admission or claims for which the total payments as paid to the insurer were less than or equal to 0 or the out-of-pocket (OOP) expenses were less than 0 (eFigure in the Supplement). We followed the Strengthening the Reporting of Observational Studies in Epidemiology (STROBE) reporting guidelines. This study qualified for exempt status from the University of Michigan’s institutional review board; data were deidentified.

### Outcomes and Variables

Our primary outcome was complications within the 90-day postoperative period. Complications were determined by the literature review and included infection, stiffness, nerve laceration, hematoma, and complex regional pain syndrome (eTable 1 in the Supplement provides *ICD-9* and *ICD-10* diagnosis codes).^9,10,11,12^ Moreover, we collected reoperation rates for treatment of infection, hematoma, or nerve injury (eTable 1 and eTable 2 in the Supplement) during the 90-day postoperative period. Encounters were collected if they had a relevant diagnosis and procedure code. We then determined the total payments and OOP expenses for the surgical episode, including anesthesia when applicable, and the 90-day postoperative period, including deductibles, copayments, and coinsurance. Total payments were defined as total facility and clinician reimbursement, including OOP expenses. All costs were inflation adjusted to the 2017 US dollar. In addition, we collected resource use data during the 90-day postoperative period. Resource use consisted of hand therapy, emergency department visits, and hospital readmissions (eTable 2 in the Supplement) that were associated with a relevant primary diagnosis or complication. The encounter types were chosen to identify use of resources beyond those in the usual global period after surgery.

We collected patient characteristics, including age, sex, median household income, insurance type, geographic region, and Elixhauser Comorbidity Index score. The Elixhauser Comorbidity Index score ranges from 0 to 19, with a higher score indicative of more comorbidities. The Elixhauser Comorbidity Index score was used as a proxy for health status of each patient using *ICD-9* and *ICD-10* diagnosis codes.^13,14^ In addition, we captured the surgical setting where the procedure was performed, which was classified as the office, HOPD, or ASC. These facility types or operative settings were established by the Centers for Medicare & Medicaid Services and are available in the IBM MarketScan.^15^

### Statistical Analysis

We calculated descriptive statistics of demographic and clinical variables by operative setting. We then performed bivariate comparisons among the operative settings and the 3 outcomes of complications, payments, and resource utilization using the χ^2^ test for categorical variables and Kruskal-Wallis test for continuous variables.

A multivariable logistic regression model was used to examine the association between complications and patient and surgical characteristics. The model was adjusted for operative setting, sex, age, median household income, insurance type, Elixhauser Comorbidity Index score, geographic region, and surgery type. We then performed 2 generalized linear regression models with log link and γ distribution to examine the proportional increase in total payments and OOP expenses. Covariates in these models included surgical location, patient age, patient sex, geographic location, procedure type, median household income, insurance type, Elixhauser Comorbidity Index score, complications, reoperation, and an interaction term for a complication requiring reoperation. In this model, the regression coefficient is a cost ratio that represents the multiplicative change in the outcome (total payments or OOP expenses) compared with the reference group. The postestimation mean marginal effects were calculated to determine the change in estimated payments relative to the reference group while adjusting for the remaining covariates. Significance level of *P* < .05 with 2-tailed, unpaired testing was set for all analyses. Statistical analyses were performed using SAS software, version 9.4 (SAS Institute Inc).

## Results

A total of 468 365 patients were included in our cohort between 2009 and 2017 with 296 378 women (63.3%) and 171 987 men (36.7%). The most common procedures performed were carpal tunnel release (252 524 [53.9%]), followed by trigger finger release (133 671 [28.5%]). Table 1 describes the study cohort characteristics, stratified by operative setting. Procedures were most commonly performed in HOPDs (284 889 [60.8%]), followed by ASCs (158 659 [33.9%]), with few (24 817 [5.3%]) performed in an office setting (*P* < .001). Trigger finger releases (13 865 [10.4%]) and excision of small hand masses (6155 [13.5%]) were more likely to be performed in the office, compared with carpal tunnel release (4145 [1.6%]) or wrist ganglion cyst excision (652 [1.8%]) (*P* < .001).

**Table 1.** Study Cohort Characteristics of 468 365 Patients Undergoing Minor Hand Surgery From 2009 to 2017

Patient characteristic	No. (%)^a^			
	Overall	Operative setting		
		Outpatient hospital	Ambulatory surgery center	Office
Total	468 365	284 889 (60.8)	158 659 (33.9)	24 817 (5.3)
Sex				
Women	296 378 (63.3)	179 886 (63.1)	101 801 (64.2)	14 691 (59.2)
Men	171 987 (36.7)	105 003 (36.9)	56 858 (35.8)	10 126 (40.8)
Age, y				
18-34	32 535 (6.9)	20 252 (7.1)	10 905 (6.9)	1378 (5.6)
35-44	55 138 (11.8)	34 471 (12.1)	18 331 (11.6)	2336 (9.4)
45-54	125 010 (26.7)	76 577 (26.9)	42 234 (26.6)	6199 (25.0)
55-64	158 582 (33.9)	94 561 (33.2)	54 671 (34.5)	9350 (37.7)
≥65	97 100 (20.7)	59 028 (20.7)	32 518 (20.5)	5554 (22.4)
Median annual household income, $				
<40 000	12 299 (2.6)	7510 (2.6)	4262 (2.7)	527 (2.1)
40 000-49 999	129 007 (27.5)	77 545 (27.2)	45 169 (28.5)	6293 (25.4)
50 000-59 999	152 514 (32.6)	90 090 (31.6)	54 620 (34.4)	7804 (31.4)
60 000-70 000	50 001 (10.7)	28 536 (10.0)	18 635 (11.7)	2830 (11.4)
>70 000	14 766 (3.2)	8005 (2.8)	5264 (3.3)	1497 (6.0)
Unspecified	109 778 (23.4)	73 203 (25.7)	30 709 (19.4)	5866 (23.6)
Insurance type				
Fee-for-service	418 800 (89.4)	254 602 (89.4)	143 530 (90.5)	20 668 (83.3)
Managed care	49 565 (10.6)	30 287 (10.6)	15 129 (9.5)	4149 (16.7)
Elixhauser Comorbidity Index score^b^				
0	252 728 (54.0)	150 929 (53.0)	87 703 (55.3)	14 096 (56.8)
1-3	40 426 (8.6)	24 824 (8.7)	13 621 (8.6)	1981 (8.0)
4-7	95 410 (20.4)	59 087 (20.7)	31 750 (20.0)	4573 (18.4)
≥8	79 801 (17.0)	50 049 (17.6)	25 585 (16.1)	4167 (16.8)
Geographic region				
Northeast	87 524 (18.7)	52 143 (18.3)	31 683 (20.0)	3698 (14.9)
North Central	130 638 (27.9)	83 969 (29.5)	40 828 (25.7)	5841 (23.5)
South	171 474 (36.6)	105 199 (36.9)	58 684 (37.0)	7591 (30.6)
West	70 155 (15.0)	37 851 (13.3)	25 519 (16.1)	6785 (27.3)
Unspecified	8574 (1.8)	5727 (2.0)	1945 (1.2)	902 (3.6)
Surgery type^c^				
Carpal tunnel release	252 524 (53.9)	165 822 (65.7)	82 557 (32.7)	4145 (1.6)
Trigger finger release	133 671 (28.5)	72 395 (54.2)	47 411 (35.5)	13 865 (10.4)
Small hand mass excision	45 597 (9.7)	23 893 (52.4)	15 549 (34.1)	6155 (13.5)
Wrist ganglion cyst excision	36 573 (7.8)	22 779 (62.3)	13 142 (35.9)	652 (1.8)

^a^ All differences significant at *P* < .001; χ^2^ testing used.

^b^ Scores range from 0 to 19, with higher scores indicating a greater number of comorbidities.

^c^ The denominators for these percentage calculations are the specific surgery types.

During the 90-day postoperative period, 15 626 patients (3.3%) incurred a complication. Patients undergoing procedures in the office had significantly fewer complications (office: 731 [2.9%], HOPD: 9718 [3.4%], and ASC: 5177 [3.3%]; *P* < .001) (Figure). Although there were statistically significant differences noted, specific types of complications and need for reoperation were clinically similar across operative settings (Figure). For example, infection occurred in 303 patients (1.2%) who underwent procedures in the office, 3637 patients (1.3%) who underwent procedures in HOPDs, and 1725 patients (1.1%) who underwent procedures in ASCs. After controlling for patient characteristics, procedures performed in HOPDs and ASCs had greater odds of having a complication compared with the office setting (HOPD: OR, 1.32; 95% CI, 1.22-1.43 and ASC: OR, 1.24; 95% CI, 1.14-1.34) (Table 2).

**Figure.**
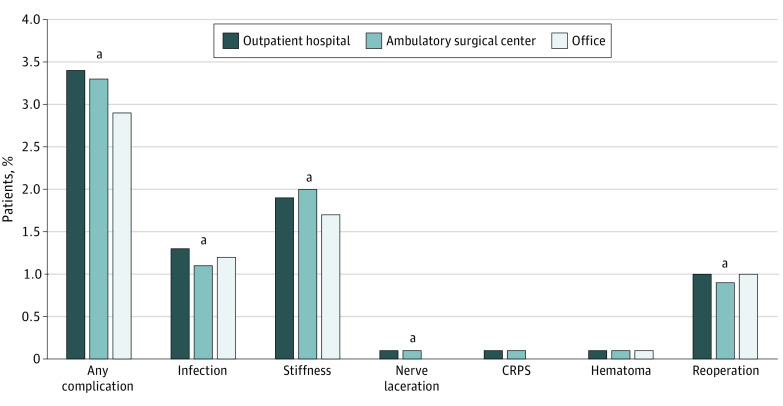
Rates of Complications and Reoperations During the 90-Day Postoperative Period After Minor Hand Surgery CRPS indicates complex regional pain syndrome. ^a^Significant at *P* < .05.

**Table 2.** Logistic Regression of Complications During the 90-Day Postoperative Period

Characteristic	Odds ratio (95% CI)
Operative setting	
Office	1 [Reference]
Outpatient hospital	1.32 (1.22-1.43)
Ambulatory surgery center	1.24 (1.14-1.34)
Sex	
Women	1 [Reference]
Men	0.98 (0.95-1.02)
Age, y	
18-34	1 [Reference]
35-44	1.04 (0.95-1.13)
45-54	1.02 (0.94-1.10)
55-64	1.03 (0.95-1.11)
≥65	1.09 (1.01-1.18)
Median annual household income, $	
<40 000	1 [Reference]
40 000-50 000	1.08 (0.97-1.19)
50 000-60 000	1.20 (1.08-1.33)
60 000-70 000	1.36 (1.21-1.53)
>70 000	1.28 (1.12-1.47)
Insurance type	
Managed care	1 [Reference]
Fee-for-service	1.15 (1.09-1.21)
Elixhauser Comorbidity Index score^a^	
0	1 [Reference]
1-3	1.26 (1.19-1.34)
4-7	1.35 (1.30-1.41)
≥8	1.60 (1.53-1.67)
Geographic region	
Northeast	1 [Reference]
North Central	1.29 (1.22-1.37)
South	1.69 (1.60-1.78)
West	1.61 (1.52-1.71)
Surgery type	
Carpal tunnel release	1 [Reference]
Trigger finger release	1.38 (1.33-1.43)
Small hand mass excision	0.96 (0.90-1.02)
Wrist ganglion cyst excision	0.75 (0.70-0.81)

^a^ Scores range from 0 to 19, with higher scores indicating a greater number of comorbidities.

Table 3 lists the total payments, OOP expenses, and resource utilization stratified by operative setting. Procedures performed in the office were significantly less expensive for payers (median total payment for surgery: $764 in the office, $1341 in HOPDs, $1202 in ASCs, *P* < .001) and patients (median OOP expenses for surgery: $41 in the office, $76 in HOPDs, $99 in ASCs, *P* < .001). Moreover, the total payment for the surgical episode plus 90-day postoperative period was $2664 (interquartile range [IQR], $1370-$4861) for procedures performed in HOPDs, $2184 (IQR, $1316-$3880) in ASCs, and $1433 (IQR, $912-$2506) in the office (*P* < .001). For OOP expenses, procedures performed in HOPDs incurred $230 (IQR, $62-$575) for the surgical episode plus 90-day postoperative period, $248 (IQR, $72-$583) in ASCs, and $152 (IQR, $44-$413) in the office. In addition, patients having office-based procedures experienced less frequent postoperative emergency department visits compared with those from the other operative settings (85 [0.3%] in the office, 1916 [0.7%] in HOPDs, and 774 [0.5%] in ASCs, *P* < .001) and lower rates of rehospitalizations (543 [2.2%] in the office, 7965 [2.8%] in HOPDs, and 3769 [2.4%] in ASCs, *P* < .001).

**Table 3.** Total Payments and Resource Use for the Surgical Episode and During the 90-Day Postoperative Period

Variable	Overall	Operative setting^a^		
		Outpatient hospital	Ambulatory surgery center	Office
Total payment, median (IQR), $				
Surgery encounter	1213 (583-2106)	1341 (553-2423)	1202 (659-1828)	764 (559-1073)
90-d postoperative period	654 (154-2199)	700 (161-2389)	612 (149-2015)	486 (123-1472)
Surgery plus 90-d postoperative period	2398 (1299-4417)	2664 (1370-4861)	2184 (1316-3880)	1433 (912-2506)
Out-of-pocket expenses, median (IQR), $				
Surgery encounter	80 (0-292)	76 (0-292)	99 (0-313)	41 (0-153)
90-d postoperative period	58 (0-212)	58 (0-216)	60 (0-210)	50 (0-176)
Surgery plus 90-d postoperative period	231 (63-569)	230 (62-575)	248 (72-583)	152 (44-413)
Resource use, No. (%)				
ED visits	2775 (0.6)	1916 (0.7)	774 (0.5)	85 (0.3)
Hand therapy	84 803 (18.1)	51 306 (18.0)	31 443 (19.8)	2054 (8.3)
Rehospitalization	12 227 (2.6)	7965 (2.8)	3769 (2.4)	543 (2.2)

Abbreviations: ED, emergency department; IQR, interquartile range.

^a^ All differences significant at *P* < .001; Kruskal-Wallis test used.

Using multivariable regression models, we determined that patients who underwent surgery in an HOPD had approximately 145% of the estimated total payment of the surgical episode plus 90-day postoperative period compared with those from the office setting (cost ratio: 1.45; 95% CI, 1.43-1.46), which conferred an additional $1216 (95% CI, $1184-$1248) (Table 4). Similarly, surgical procedures performed in ASCs were approximately 126% of the estimated total payment of office procedures (cost ratio: 1.26; 95% CI, 1.25-1.27), corresponding to an added $709 (95% CI, $676-$741).

**Table 4.** Generalized Linear Regression of Total Payments and Out-of-Pocket Expenses for Surgery Plus 90-Day Postoperative Period

Characteristic	Total payment		Out-of-pocket expenses	
	Cost ratio (95% CI)	$ Change (95% CI)^a^	Cost ratio (95% CI)	$ Change (95% CI)^a^
Operative setting				
Office	1 [Reference]		1 [Reference]	
Outpatient hospital	1.45 (1.43-1.46)	1216 (1184-1248)	1.29 (1.27-1.31)	115 (109-121)
Ambulatory surgery center	1.26 (1.25-1.27)	709 (676-741)	1.35 (1.34-1.37)	140 (134-146)
Sex				
Women	1 [Reference]		1 [Reference]	
Men	0.99 (0.99-1.00)	−36 (−55 to −17)	1.00 (0.99-1.00)	−3 (−6 to 1)
Age, y				
18-34	1 [Reference]		1 [Reference]	
35-44	1.05 (1.04-1.07)	189 (146-231)	0.96 (0.95-0.98)	−22 (−31 to −13)
45-54	1.07 (1.06-1.08)	241 (203-280)	0.95 (0.93-0.96)	−33 (−41 to −25)
55-64	1.09 (1.08-1.10)	306 (268-344)	0.91 (0.90-0.92)	−55 (−63 to −46)
≥65	1.00 (0.99-1.01)	7 (−33 to 48)	0.52 (0.51-0.52)	−286 (−294 to −278)
Median annual household income, $				
<40 000	1 [Reference]		1 [Reference]	
40 000-50 000	1.00 (0.98-1.01)	−9 (−63 to 46)	1.03 (1.01-1.06)	14 (6-23)
50 000-60 000	1.07 (1.06-1.09)	251 (196-306)	1.05 (1.03-1.08)	28 (19-37)
60 000-70 000	1.16 (1.14-1.18)	563 (500-627)	1.14 (1.12-1.17)	47 (37-58)
>70 000	1.24 (1.22-1.27)	843 (762-924)	1.03 (1.00-1.06)	22 (9-35)
Geographic region				
Northeast	1 [Reference]		1 [Reference]	
North Central	0.96 (0.95-0.97)	−161 (−192 to 130)	1.32 (1.31-1.33)	125 (121-130)
South	0.88 (0.87-0.89)	−471 (−500 to −441)	1.40 (1.38-1.41)	142 (138-146)
West	1.00 (0.99-1.01)	−7 (−42 to 27)	1.35 (1.33-1.36)	150 (145-156)
Elixhauser Comorbidity Index score^b^				
0	1 [Reference]		1 [Reference]	
1-3	1.23 (1.21-1.24)	728 (693-762)	0.98 (0.96-0.99)	−14 (−20 to −8)
4-7	1.22 (1.22-1.23)	724 (700-748)	0.97 (0.96-0.98)	−19 (−23 to −15)
≥8	1.49 (1.48-1.50)	1585 (1554-1616)	1.00 (0.99-1.01)	−10 (−15 to −5)
Insurance type				
Managed care	1 [Reference]		1 [Reference]	
Fee-for-service	1.09 (1.08-1.10)	321 (293-349)	1.62 (1.60-1.64)	206 (203-210)
Surgery type				
Carpal tunnel release	1 [Reference]		1 [Reference]	
Trigger finger release	0.85 (0.85-0.86)	−609 (−630 to −587)	0.94 (0.93-0.94)	−35 (−39 to −31)
Small hand mass excision	0.65 (0.64-0.65)	−1456 (−1481 to −1430)	0.76 (0.75-0.76)	−130 (−135 to −126)
Wrist ganglion cyst excision	0.74 (0.74-0.75)	−1057 (−1088 to −1026)	0.86 (0.85-0.87)	−71 (−76 to −66)
Complication				
No complication	1 [Reference]		1 [Reference]	
With reoperation	1.50 (1.42-1.58)	2815 (2202-3428)	1.25 (1.16-1.33)	114 (98-130)
Without reoperation	1.33 (1.31-1.35)	1067 (1001-1134)	1.19 (1.16-1.21)	80 (70-91)
Reoperation				
No reoperation	1 [Reference]		1 [Reference]	
With a specified complication	1.70 (1.63-1.78)	3018 (2372-3664)	1.21 (1.14-1.28)	112 (83-140)
Without a specified complication	1.51 (1.46-1.56)	1270 (1179-1362)	1.15 (1.11-1.20)	78 (58-97)

^a^ Obtained using postestimation average marginal effects. Value represents increase in mean predicted total payment or out-of-pocket expenses relative to the reference group.

^b^ Scores range from 0 to 19, with higher scores indicating a greater number of comorbidities.

In the adjusted regression for OOP expenses, minor procedures performed in HOPDs were 129% (cost ratio: 1.29; 95% CI, 1.27-1.31) of the estimated OOP expenses of procedures performed in the office, which conferred an additional $115 in OOP expenses (95% CI, $109-$121) (Table 4). Procedures performed in ASCs were 135% (cost ratio: 1.35; 95% CI, 1.34-1.37) of the estimated OOP expenses of office procedures, leading to an extra $140 in OOP expenses (95% CI, $134-$146). Shifting ASC and HOPD procedures to the office could save patients an estimated $6 million annually during the study period in OOP expenses.

## Discussion

In this nationwide analysis of 468 365 minor hand procedures, office-based procedures were safe in terms of complication rates relative to ambulatory and hospital-based operative settings. Procedures performed in the office incurred lower payments for payers and patients. Predictably, complications and reoperations were associated with increased payments for both payers and patients, regardless of operative setting. Shifting minor procedures to the office may reduce health care costs as a whole and decrease the financial burden placed on patients without compromising care quality.

Debate exists regarding the safety of office-based procedures. This debate may be because of the unregulated nature of office-based procedures with little federal oversight. In a study of all surgical procedures performed in Florida during a 2-year period, Vila et al^16^ found a 10-fold increase in adverse incidents among procedures performed in office settings compared with ambulatory surgery centers. The Vila study^16^ included a range of surgical procedures from general surgery to cosmetic surgery and did not account for type of procedure in the analysis. In the present study of minor-complexity procedures, the complication rates in the 3 operative settings were clinically similar, ranging from 2.9% to 3.5%, providing reassurance that minor procedures performed in the office were not associated with higher complication rates. After adjusting for patient characteristics, minor hand surgery procedures performed in hospital- or ambulatory-based operative settings had significantly higher odds of a complication compared with the office setting. These findings build on previous work assessing outcomes of other minor procedures performed in the office setting. In a study of over 13 500 cataract procedures, Ianchulev et al^17^ reported a similar safety profile in office-based cataract surgery compared with procedures performed in ASCs and HOPDs. Another study investigating ureteral stent placement in the office found no statistically significant differences in failure of stent placement and in stent-related complications compared with stents placed under general anesthesia.^18^ In addition, a study by Wortman et al^19^ of office-based hysteroscopy revealed that 98.8% of patients were highly satisfied with their procedure, concluding that procedures can be performed in the office with a high degree of patient satisfaction. For specific surgical procedures, the office setting is a safe alternative to hospital- and ambulatory-based facilities.

Operative setting has a substantial association with spending for the payer and the patient. Data from the Centers for Medicare & Medicaid Services have shown an increase in outpatient surgery from 2008 to 2010 with a transition to freestanding ASCs.^20^ Because of this transition and concerns about increased spending, the Centers for Medicare & Medicaid Services have enacted policies to curb ASC spending with mixed efficacy.^21^ In a study by Carey,^22^ HOPDs received lower payments for similar surgical procedures in areas with a high density of ASCs, suggesting an influence of ASCs on price negotiation. However, few studies have addressed the spending associated with procedures that can be performed in multiple operative settings, specifically the office setting. In a single-institution study by Rhee et al,^1^ performing minor hand procedures in the office saved approximately $393 100 compared with performing similar procedures in the operating room. Moreover, in a national study of carpal tunnel releases, the largest contributor to greater charges was operative setting, with HOPDs associated with a $500 higher charge than ASCs.^23^ However, the office setting was not included in this analysis. In our study, only 5.3% of minor procedures were performed in the office setting, but these office-based procedures were significantly less costly than those performed in other operative settings, highlighting the underuse of the office as an operative setting. Although the difference in cost associated with operative setting is not surprising, it raises the question as to why more minor procedures are not being transitioned to the office setting. Currently, payer and hospital incentives are lacking to encourage office-based procedures. In addition, there may be clinician-level barriers to performing surgery in the office, such as disruption of clinical workflow, need to secure and process surgical instruments, and lack of technical assistants. Patient preference may contribute to these barriers. Despite these hurdles, policies to incentivize clinicians and hospitals to overcome these challenges and perform procedures in less costly settings may be one avenue to decrease overall health care spending.

Research has shown that patients are bearing more of the financial burden of health care through increasing deductibles, coinsurance, and copayments.^24^ However, patients rarely know their OOP expenses before using health care services, and there continues to be a lack of transparency in what patients will have to pay for rendered services. This is especially true for facility fees that can vary substantially based on operative setting, even for a seemingly minor procedure. In addition, patients and clinicians have a limited grasp of OOP spending, which may lead to financial harm for patients.^25,26,27,28^ This study corroborates these concerns, with a potential savings of over $6 million annually in OOP expenses for the study cohort patients if they received their minor hand surgical care in the office rather than the HOPDs and ASCs. Given the financial burden of OOP expenses for surgical procedures, clinicians should consider the operative setting as a modifiable cost contributor for minor procedures, which may decrease the potential financial harm to patients.

### Limitations

This study has inherent limitations of insurance claims data. The IBM MarketScan databases lack granular clinical data. For example, these databases do not contain severity of disease, which may affect choice in operative setting. Also, we could not assess specific reoperation rates because of the unspecified nature of *Current Procedural Terminology* codes, such as redo carpal tunnel release or redo excision of ganglion cysts. *Current Procedural Terminology* codes do not describe the specifics of which finger or hand had the initial operation. In addition, we do not know the rationale for choice of operative setting, which may influence complication rates. Clinician and patient preference may influence decision-making on operative setting. There may also be selection bias, with patients who have more complex conditions undergoing procedures in HOPDs and ASCs; however, the Elixhauser Comorbidity Index scores among the operative settings were clinically similar, revealing comparable health status among the patients. In addition, the procedures chosen typically have limited variation in procedural complexity. Clinician characteristics, including surgical skill or experience, may influence choice of operative setting and subsequent complication rates, which cannot be assessed using the IBM MarketScan databases. This study used diagnosis and procedure codes that are for billing, which may be subject to coding inaccuracies. However, coding inaccuracies of minor hand procedure complications are unlikely to differ across operative settings. The IBM MarketScan databases include patients from a large, commercially insured population, thus limiting the generalizability of this study to uninsured patients, patients with Medicare, and patients with Medicaid. In addition, this analysis only assessed payments, complications, and resource use during the 90-day postoperative period and did not include potential longer-term complications and subsequent costs.

## Conclusions

This nationwide analysis appears to support performing minor procedures in the office setting. Office-based procedures were safe and had lower total payments and OOP expenses compared with procedures performed in hospital- and ambulatory-based settings. Shifting minor procedures from HOPDs and ASCs to the office setting could meaningfully influence spending for clinicians and patients without compromising patient safety. Policies are needed to incentivize clinicians and health systems to perform minor procedures in the office setting to encourage them to overcome implementation challenges and help minimize unnecessary health care costs for patients.

## Notes

Supplement. **eTable 1.** Diagnosis Codes of Interest **eTable 2.** Procedure Codes and Healthcare Common Procedure Codes of Interest **eFigure.** Inclusion and Exclusion Criteria
