# Supplementary material for: Comparison of Safety and Insurance Payments for Minor Hand Procedures Across Operative Settings
Source: JAMA Netw Open. 2020 Oct 13;3(10):e2015951. doi: 10.1001/jamanetworkopen.2020.15951 (PMC8094424; doi:10.1001/jamanetworkopen.2020.15951)
Supplement: Supplement. — eTable 1. Diagnosis Codes of Interest eTable 2. Procedure Codes and Healthcare Common Procedure Codes of Interest eFigure. Inclusion and Exclusion Criteria [file jamanetwopen-e2015951-s001.pdf]

Supplementary Online Content

Billig JI, Nasser JS, Chen JS, et al. Comparison of safety and insurance payments for minor hand procedures across operative settings. *JAMA Netw Open*. 2020;3(10):e2015951. doi:10.1001/jamanetworkopen.2020.15951

eTable 1. Diagnosis Codes of Interest

eTable 2. Procedure Codes and Healthcare Common Procedure Codes of Interest

eFigure. Inclusion and Exclusion Criteria

This supplementary material has been provided by the authors to give readers additional information about their work.

© 2020 Billig JI et al. *JAMA Network Open.*

eTable 1: Diagnosis Codes of Interest

**ICD-9/10* Codes of Interest**		
**Primary Diagnoses**		
Benign soft tissue masses		
782.2	ICD-9	Localized superficial swelling, mass or lump
R22.3X	ICD-10	Localized swelling, mass and lump of skin and subcutaneous tissue, upper limb
214.1	ICD-9	Lipoma, skin
215.2	ICD-9	Benign soft tissue tumor, shoulder or distal
D17.21	ICD-10	Benign lipomatous neoplasm of skin and subcutaneous tissue of right arm
D17.22	ICD-10	Benign lipomatous neoplasm of skin and subcutaneous tissue of left arm
228.0	ICD-9	Hemangioma, lymphangioma, or glomus tumor, any site
D18.01	ICD-10	Hemangioma of skin and subcutaneous tissue
D21.1X	ICD-10	Benign neoplasm of connective and other soft tissue of upper limb, including shoulder
228.01	ICD-9	Vascular skin tumor, including glomus tumor
228.1	ICD-9	Lymphangioma, any site
D18.1	ICD-10	Lymphangioma, any site
686.1	ICD-9	Pyogenic granuloma
L98.0	ICD-10	Pyogenic granuloma
706.2	ICD-9	Sebaceous cyst
709.4	ICD-9	Foreign body granuloma, skin
727.02	ICD-9	Giant cell tumor of tendon sheath
Carpal Tunnel Syndrome and Related Symptoms		
354.0	ICD-9	Carpal Tunnel Syndrome
354.1	ICD-9	Other lesion of median nerve
G56.0X	ICD-10	Carpal Tunnel Syndrome
G56.1X	ICD-10	Other lesion of median nerve
Ganglion		
727.42	ICD-9	Ganglion of tendon sheath
727.41	ICD-9	Ganglion of joint
M67.43X	ICD-10	Ganglion, wrist
M67.44X	ICD-10	Ganglion, hand
Trigger Finger		
727.03	ICD-9	Trigger finger
719.24	ICD-9	Synovitis hand
M65.3X	ICD-10	Trigger finger
**Complications**		
Complex Regional Pain Syndrome		
337.21	ICD-9	Reflex sympathetic dystrophy, upper limb
G56.4X	ICD-10	Causalgia of upper limb

© 2020 Billig JI et al. *JAMA Network Open.*

G90.51	ICD-10	Complex regional pain syndrome I of upper limb
Hematoma		
998.11	ICD-9	Hemorrhage complicating a procedure
998.12	ICD-9	Hematoma complicating a procedure
L76.0X	ICD-10	Intraoperative hemorrhage and hematoma of skin and subcutaneous tissue complicating a procedure
L76.2X	ICD-10	Postprocedural hemorrhage of skin and subcutaneous tissue following a procedure
L76.3X	ICD-10	Postprocedural hematoma and seroma of skin and subcutaneous tissue following a procedure
Infection		
682.9	ICD-9	Cellulitis and abscess of unspecified sites
711.00	ICD-9	Pyogenic arthritis, site unspecified
711.04	ICD-9	Pyogenic arthritis, hand
711.90	ICD-9	Unspecified infective arthritis, site unspecified
711.94	ICD-9	Unspecified infective arthritis, hand
730.94	ICD-9	Unspecified infection of hand
998.51	ICD-9	Infected postoperative seroma
998.59	ICD-9	Other postoperative infection
L02.413	ICD-10	Cutaneous abscess of right upper limb
L02.414	ICD-10	Cutaneous abscess of left upper limb
L02.51X	ICD-10	Cutaneous abscess of hand
L02.91	ICD-10	Cutaneous abscess, unspecified
L03.01X	ICD-10	Cellulitis of finger
L03.02X	ICD-10	Acute lymphangitis of finger
L03.113	ICD-10	Cellulitis of right upper limb
L03.114	ICD-10	Cellulitis of left upper limb
L03.123	ICD-10	Acute lymphangitis of right upper limb
L03.124	ICD-10	Acute lymphangitis of left upper limb
L03.90	ICD-10	Cellulitis unspecified
L03.91	ICD-10	Acute lymphangitis unspecified
L76.0	ICD-10	Intraoperative hemorrhage and hematoma of skin and subcutaneous tissue complicating a procedure
L76.2	ICD-10	Postprocedural hemorrhage of skin and subcutaneous tissue following a procedure
L76.3	ICD-10	Postprocedural hematoma and seroma of skin and subcutaneous tissue following a procedure
L76.8	ICD-10	Other intraoperative and postprocedural complications of skin and subcutaneous tissue
M00.00	ICD-10	Staphylococcus arthritis, unspecified joint
M00.03	ICD-10	Staphylococcus arthritis, wrist
M00.04	ICD-10	Staphylococcus arthritis, hand
M00.00	ICD-10	Pneumococcal arthritis, unspecified joint
M00.13X	ICD-10	Pneumococcal arthritis, wrist

M00.14X	ICD-10	Pneumococcal arthritis, hand
M00.20	ICD-10	Other streptococcal arthritis, unspecified joint
M00.23X	ICD-10	Other streptococcal arthritis, wrist
M00.24X	ICD-10	Other streptococcal arthritis, hand
M00.80	ICD-10	Arthritis due to other bacteria, unspecified joint
M00.83X	ICD-10	Arthritis due to other bacteria, wrist
M00.84X	ICD-10	Arthritis due to other bacteria, hand
M00.9	ICD-10	Pyogenic arthritis, unspecified
M65.03X	ICD-10	Abscess of tendon sheath, forearm
M65.04X	ICD-10	Abscess of tendon sheath, hand
M65.13X	ICD-10	Other infective (teno)synovitis, wrist
M65.14X	ICD-10	Other infective (teno)synovitis, hand
M71.03X	ICD-10	Abscess of bursa, wrist
M71.04X	ICD-10	Abscess of bursa, hand
M71.13X	ICD-10	Other infective bursitis, wrist
M71.14X	ICD-10	Other infective bursitis, hand
Nerve laceration		
955.6	ICD-9	Injury to digital nerve, upper limb
S64.3X	ICD-10	Injury to digital nerve of thumb
S64.4X	ICD-10	Injury of digital nerve of other and unspecified finger
955.1	ICD-9	Injury to median nerve
955.5	ICD-9	Injury to cutaneous sensory nerve, upper limb
S64.1X	ICD-10	Injury of median nerve at wrist and hand level
S64.8X	ICD-10	Injury of other nerves at wrist and hand level
Stiffness		
718.44	ICD-9	Contracture of joint, hand
718.54	ICD-9	Ankylosis of joint, hand
719.54	ICD-9	Stiffness of joint, NOS, hand
M24.53X	ICD-10	Contracture, wrist
M24.54X	ICD-10	Contracture, hand
M24.63X	ICD-10	Ankylosis, wrist
M24.64X	ICD-10	Ankylosis, hand
M24.83	ICD-10	Other specific joint derangement of wrist, NOS
M24.84X	ICD-10	Other specific joint derangements of hand, NOS
M25.63X	ICD-10	Stiffness of wrist, NOS
M25.64X	ICD-10	Stiffness of hand, NOS

*ICD-9: International Classification of Diseases, Ninth Revision; ICD-10: International Statistical Classification of Diseases and Related Health Problems, Tenth Revision

© 2020 Billig JI et al. *JAMA Network Open.*

eTable 2: Procedure Codes and Healthcare Common Procedure Codes of Interest

**CPT and HCPCS Codes**		
Carpal Tunnel Syndrome and Related Symptoms		
64721	CPT	Open carpal tunnel release
Ganglion Excision		
25111	CPT	Excision of ganglion, wrist (dorsal or volar)
Benign Soft Tissue Mass Excision		
26116	CPT	Excision of tumor or vascular malformation, hand or finger, deep, subfascial, intramuscular
25075	CPT	Excision, tumor, forearm and/or wrist area, subcutaneous
25076	CPT	Excision, tumor, forearm, and/or wrist area, subfascial or intramuscular
26115	CPT	Excision, tumor or vascular malformation, hand or finger, subcutaneous
26160	CPT	Excision of lesion of tendon sheath or capsule (cyst, mucous cyst, or ganglion), hand or finger
Trigger Finger		
26055	CPT	Trigger finger release
**Reoperations**		
Infection and Hematoma		
10060	CPT	Incision and drainage of abscess, simple
10061	CPT	Incision and drainage of abscess, complicated or multiple
10140	CPT	Incision and drainage of hematoma, seroma, or fluid collection
10160	CPT	Puncture aspiration of abscess, hematoma, bulla or cyst
10180	CPT	Incision and drainage, complex, postoperative wound infection
11000	CPT	Debridement of infected skin, up to 10% of total body surface area
11001	CPT	Debridement of infected skin, each additional 10%
11040	CPT	Debridement, skin partial thickness
11041	CPT	Debridement skin, full thickness
11042	CPT	Debridement skin, subcutaneous tissue
11043	CPT	Debridement skin, subcutaneous tissue, and muscle
20000	CPT	Incision of soft tissue abscess, superficial
20005	CPT	Incision of soft tissue abscess, deep or complicated
25028	CPT	Incision and drainage, forearm and/or wrist, deep abscess or hematoma
25031	CPT	Incision and drainage, forearm and/or wrist, infected bursa
25035	CPT	Incision, deep, with opening of bone cortex, forearm and/or wrist
26010	CPT	Drainage of finger abscess, simple
26011	CPT	Drainage of finger abscess, complex
26020	CPT	Drainage of tendon sheath, one digit and/or palm
26025	CPT	Drainage of palmar bursa, single, ulnar or radial

© 2020 Billig JI et al. *JAMA Network Open.*

26030	CPT	Drainage of palmar bursa, multiple or complicated
Nerve Injury		
64831	CPT	Suture of digital nerve, hand or foot, one nerve
64832	CPT	Suture of digital nerve, hand or foot, each additional
64835	CPT	Suture of 1 nerve hand or foot, median motor thenar
64857	CPT	Suture of major peripheral nerve arm or leg, except sciatic without transposition
**Resource Utilization**		
Emergency Department Visits		
99281	CPT	ED visit I
99282	CPT	ED visit II
99283	CPT	ED visit III
99284	CPT	ED visit IV
99285	CPT	ED visit V
Occupational therapy		
97001	CPT	Physical therapy evaluation
97002	CPT	Physical therapy re-evaluation
97003	CPT	Occupational therapy evaluation
97004	CPT	Occupational therapy re-evaluation
98960	CPT	Education and training for patient self-management by a qualified
97035	CPT	Ultrasound therapy
97010	CPT	Application of modality to 1 or more areas; hot or cold packs
97012	CPT	Traction, mechanical
97014	CPT	Application of a modality to 1 or more areas; electrical stimulation (unattended)
97018	CPT	Application of a modality to 1 or more areas; paraffin bath
97022	CPT	Application of a modality to 1 or more areas; whirlpool
97026	CPT	Infrared
97032	CPT	Application of a modality to 1 or more areas; electrical stimulation (manual)
97033	CPT	Application of a modality to 1 or more areas; iontophoresis
97110	CPT	Therapeutic exercises
97112	CPT	Neuromuscular re-education
97124	CPT	Massage therapy
97140	CPT	Manual therapy
97530	CPT	Therapeutic activities
90901	CPT	Biofeedback training by any modality
97039	CPT	Unlisted modality (specify type and time if constant attendance)
97760	CPT	Orthotic(s) management and training (including assessment and fitting when not otherwise reported)
97762	CPT	Checkout for orthotic/prosthetic use
29125	CPT	Application of short arm splint (forearm to hand); static

© 2020 Billig JI et al. *JAMA Network Open.*

29126	CPT	Application of short arm splint (forearm to hand); dynamic
A4570	HCPCS	Splint
L3807	HCPCS	Wrist hand finger orthosis, without joint(s), prefabricated item that has been trimmed, bent, molded, assembled, or otherwise customized to fit a specific patient by an individual with expertise
L3908	HCPCS	Wrist hand orthosis, wrist extension control cock-up, non-molded, prefabricated, off-the-shelf
A4590	HCPCS	Special casting material
L3806	HCPCS	Wrist hand finger orthosis without joint custom fabricated
L3809	HCPCS	Wrist hand finger orthosis without joint pre-fabricated
L3900	HCPCS	Hinge extension/flex wrist/f
L3908	HCPCS	Wrist hand orthosis, cock-up non-molded pre-fabricated
L3913	HCPCS	Hand finger orthosis without joints custom fabricated
L3915	HCPCS	Wrist hand orthosis nontorsion joints pre-fabricated
L3919	HCPCS	Hand orthosis without joints custom fabricated
L3923	HCPCS	Hand finger orthosis without joints pre-fabricated
L3999	HCPCS	Upper limb orthosis, not otherwise specified
L4210	HCPCS	Orthotic device repair/replace minor parts
Q4051	HCPCS	Splint supplies miscellaneous

CPT: Current Procedural Code; HCPCS: Healthcare Common Procedure Coding System

© 2020 Billig JI et al. *JAMA Network Open.*

eFigure: Inclusion and Exclusion Criteria

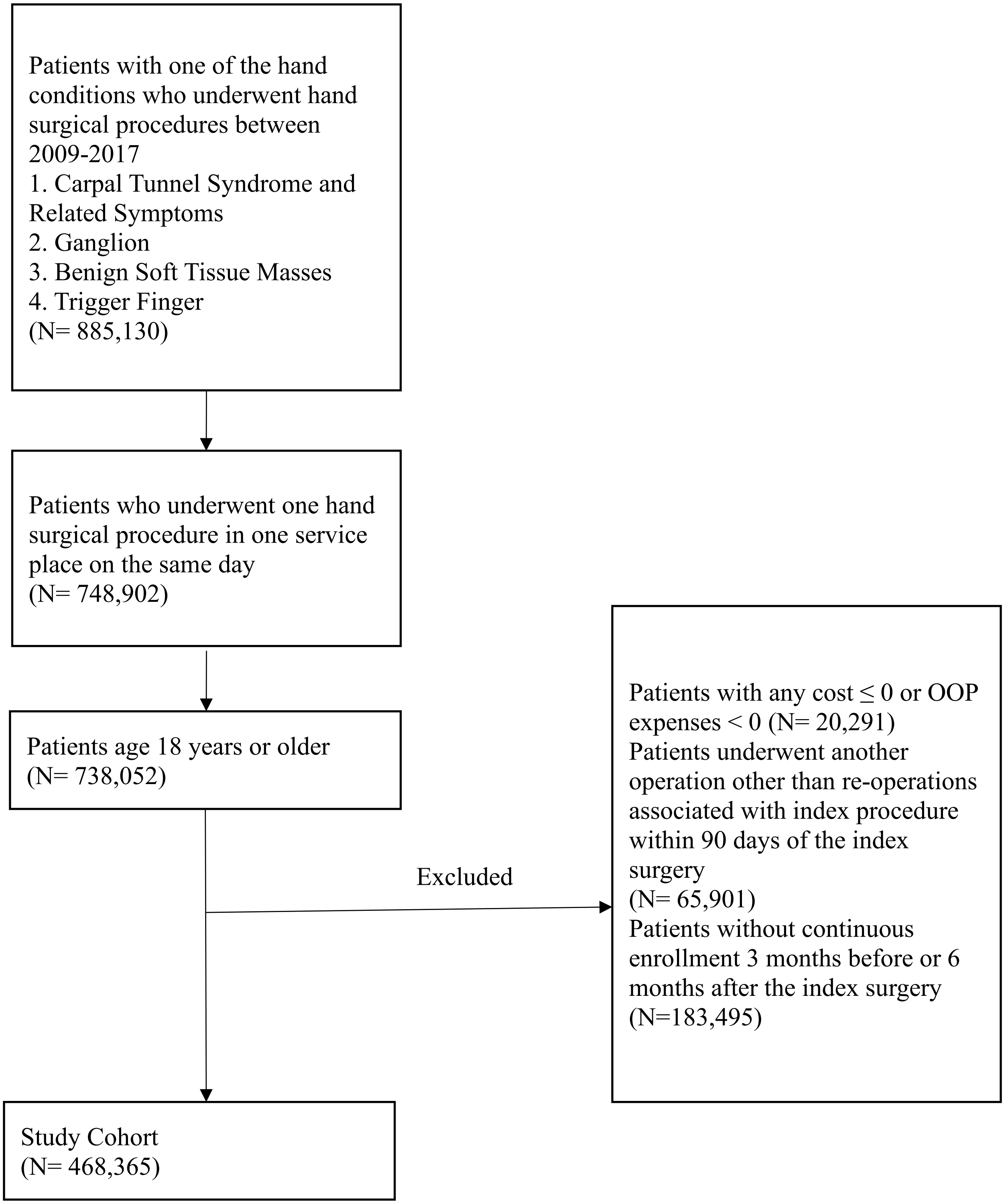

OOP: Out-of-pocket

© 2020 Billig JI et al. *JAMA Network Open.*
